# Abdominal Pain, the Adolescent and Altered Brain Structure and Function

**DOI:** 10.1371/journal.pone.0156545

**Published:** 2016-05-31

**Authors:** Catherine S. Hubbard, Lino Becerra, Nicole Heinz, Allison Ludwick, Tali Rasooly, Rina Wu, Adriana Johnson, Neil L. Schechter, David Borsook, Samuel Nurko

**Affiliations:** 1 Center for Pain and the Brain, Boston Children’s Hospital, Waltham, Massachusetts, United States of America; 2 Department of Anesthesiology, Perioperative and Pain Medicine, Boston Children’s Hospital, Boston, Massachusetts, United States of America; 3 Department of Anaesthesia, Harvard Medical School, Boston, Massachusetts, United States of America; 4 Center for Motility and Functional Gastrointestinal Disorders, Division of Gastroenterology, Department of Medicine, Boston Children’s Hospital, Boston, Massachusetts, United States of America; Institute of Psychology, Chinese Academy of Sciences, CHINA

## Abstract

Irritable bowel syndrome (IBS) is a functional gastrointestinal (GI) disorder of unknown etiology. Although relatively common in children, how this condition affects brain structure and function in a pediatric population remains unclear. Here, we investigate brain changes in adolescents with IBS and healthy controls. Imaging was performed with a Siemens 3 Tesla Trio Tim MRI scanner equipped with a 32-channel head coil. A high-resolution T1-weighted anatomical scan was acquired followed by a T2-weighted functional scan. We used a surface-based morphometric approach along with a seed-based resting-state functional connectivity (RS-FC) analysis to determine if groups differed in cortical thickness and whether areas showing structural differences also showed abnormal RS-FC patterns. Patients completed the Abdominal Pain Index and the GI Module of the Pediatric Quality of Life Inventory to assess abdominal pain severity and impact of GI symptoms on health-related quality of life (HRQOL). Disease duration and pain intensity were also assessed. Pediatric IBS patients, relative to controls, showed cortical thickening in the posterior cingulate (PCC), whereas cortical thinning in posterior parietal and prefrontal areas were found, including the dorsolateral prefrontal cortex (DLPFC). In patients, abdominal pain severity was related to cortical thickening in the intra-abdominal area of the primary somatosensory cortex (SI), whereas HRQOL was associated with insular cortical thinning. Disease severity measures correlated with cortical thickness in bilateral DLPFC and orbitofrontal cortex. Patients also showed reduced anti-correlations between PCC and DLPFC compared to controls, a finding that may reflect aberrant connectivity between default mode and cognitive control networks. We are the first to demonstrate concomitant structural and functional brain changes associated with abdominal pain severity, HRQOL related to GI-specific symptoms, and disease-specific measures in adolescents with IBS. It is possible such changes will be responsive to therapeutic intervention and may be useful as potential markers of disease progression or reversal.

## Introduction

Irritable bowel syndrome (IBS) is a functional gastrointestinal disorder (FGID) characterized by chronic abdominal pain and/or discomfort, and accompanied by altered bowel patterns [1, 2]. Although more frequently studied and reported in adults, this syndrome also affects children and adolescents and is associated with the occurrence of early, adverse life events, and the development of mood and somatoform disorders into adulthood [3–5]. In Western societies alone, the reported incidence of pediatric IBS is not uncommon, with an estimated 8–23% diagnosed between 4 and 18 years (yrs.) of age [6, 7]. Furthermore, the presentation of IBS symptoms can be debilitating for a child, interfering with school and other social activities, leading to impairments in daily functioning and decreasing overall well-being and quality of life [8, 9]. Therefore, IBS represents an important clinical problem in children that needs to be addressed since identifying effective treatments for this syndrome would be life-altering for many.

IBS is considered a ‘functional disorder’ given that alterations in bowel function and associated symptoms manifest in absence of any apparent structural or biochemical disease process. Despite the fact that IBS diagnosis has evolved from one entirely based on exclusion to a symptom-based approach currently embodied by Rome III criteria [10, 11], diagnosis still remains descriptive and without a definitive or objective biomarker. Undoubtedly, the availability of such markers would form a basis of enhanced phenotyping of the disease state, provide an earlier diagnosis, reduce costs associated with unnecessary and expensive testing, and provide valuable information to researchers and clinicians in search of novel and effective treatments.

To date, the pathophysiology of IBS is not well understood. The etiology of this syndrome is considered multifactorial, with central sensitization a likely contributing factor [12–15]. A number of putative mechanisms have been described in an attempt to explain the neurobiology underlying IBS, including disruptions in gut-brain axis signaling [12, 14, 16]; a hypothesis now made more tractable to scientific inquiry with the advent of magnetic resonance imaging (MRI). Collectively, neuroimaging studies performed in adults with IBS have reported structural and functional brain changes that were associated with (i) increased visceral sensitivity [17]; (ii) altered affective processes that modulate visceral pain [18]; and (iii) disruptions in endogenous descending pain inhibitory mechanisms [19, 20]. In contrast to the adult literature, our understanding of how IBS affects brain structure and function in children is less well delineated. Furthermore, while an IBS diagnosis in children shares similar diagnostic criteria to that in adults, the early structural and functional brain changes associated with the onset and progression of this syndrome may differ dramatically in the developing brain and warrants investigation.

The primary aim of this study was to identify concurrent structural and functional brain changes in adolescents with a clinical diagnosis of IBS relative to healthy controls using MRI. A secondary aim was to examine whether alterations in brain morphology were related to abdominal pain severity, health-related quality of life (HRQOL) as it pertains to GI-specific symptoms, and clinical measures of disease duration and pain intensity (over the past week). Specifically, we asked the following questions: (1) Are there significant structural and functional changes in the brains of pediatric patients with IBS compared to healthy cohorts? (2) If so, do these brain changes in adolescents with IBS parallel those reported in adults? and lastly, (3) To what extent are these changes associated with the severity of current abdominal pain, impact of GI-related symptoms on health and well-being, and clinical variables such as disease duration and retrospective pain intensity ratings?

## Materials and Methods

### Participants

Nineteen adolescents (mean age ± SD = 16.00 ± 2.09; Females = 15) diagnosed with IBS (based on Rome III criteria; see below) were prospectively enrolled in this study. All patients were frequency matched to healthy controls on the basis of age and gender (see Results section for final sample demographics). For the healthy controls, all demographic, psychometric, and neuroimaging data were obtained from our existing P.A.I.N. Group database. In both groups, scanning parameters and procedures for image acquisition were identical and carried out using the same scanner. All other study-related procedures were the same with the exception of psychometric questionnaire administration; only psychometric questionnaire data were available for the patient group.

General inclusion criteria required that all participants were right-handed, between the age of 8 and 21 yrs., had no indication of claustrophobia or suicidal ideation, passed MRI safety screening, and had negative urine tests for drugs of abuse and/or pregnancy. In addition, healthy controls were deemed eligible for inclusion in the current study as long as they reported no history of a psychiatric disorder, neurological disease, or serious medical condition and were not currently taking any medications with CNS effects. Prior to commencement of study-related procedures, written informed consent or assent was given by each participant (with parental permission for children < 18 yrs.). All study-related procedures and materials were approved by the Boston Children’s Hospital (BCH) Institutional Review Board (IRB-P00007161 initial approval date was 10/02/2013) and conducted in accordance with the Declaration of Helsinki.

Pediatric patients with IBS were recruited from the Motility and Functional Gastrointestinal Disorders Center and the General GI Clinics at BCH. Diagnoses were made by a gastroenterologist (S.N.) using pediatric Rome III criteria [11, 21]. All bowel habit subtypes were included in this study. Exclusionary factors for the patient group included any evidence of organic gastrointestinal disease, hepatic disorders, positive laboratory test results including abnormalities in complete blood count, erythrocyte sedimentation rate, albumin, serum amylase, lipase, liver enzymes, urine analysis, celiac screening, and stool examination for occult blood. Exclusion based on medication usage in patients was determined on a case-by-case basis. Proton pump inhibitors and antispasmodics were allowed as long as patient had been on a stable dose for at least 4 weeks. In general, patients were never asked to refrain from taking their prescribed medication and in some cases, were included if they were on a stable (at least 3 months), low to moderate dose of a psychotropic medication (e.g., anti-depressant or anxiolytics).

### Psychometric and Clinical Measures

All patients fulfilled diagnostic criteria for IBS using The Questionnaire on Pediatric Gastrointestinal Symptoms—Rome III Version [21]. In addition, patients completed a series of self-report questionnaires to assess characteristics of their abdominal pain severity, impact of GI-related symptoms on HRQOL, extent of functional disability, mood disturbances and degree of pain catastrophizing. Questionnaires administered included The Abdominal Pain Index (API) [22, 23], the Pediatric Quality of Life Inventory (PedsQL; version 4.0) (Child report, ages 8–12; Teen Report, ages 13–18; Young Adult Report, ages 18–25) [24], the PedsQL Gastrointestinal (GI) Symptoms Module (PedsQL GI module; version 3.0) [25] and the Functional Disability Inventory (FDI) [26]. Other self-report measures included the Revised Children’s Anxiety and Depression Scale (RCADS) [27], and the Pain Catastrophizing Scale−Child Version (PCS-C) [28].

The API was used to provide an overall measure of abdominal pain severity and consisted of five items assessing the frequency, duration, and intensity of abdominal pain in the past two weeks. The PedsQL is a 23-item questionnaire, using a 5-point Likert scale (0 = never a problem, 1 = almost never a problem, 2 = sometime a problem, 3 = often a problem, 4 = almost always a problem), that measures the core dimensions of health and well-being in terms of physical (8-items), emotional (5-items), social (5-items) and school functioning (5-items). This measure yields a PedsQL total score with lower scores representing greater GI related symptoms and decreased health-related quality of life. The PedsQL GI Symptoms Module contains 74-items from 14 different scales describing both GI and non-GI related symptoms. All items on this measure, like the core PedsQL, use the same 5-point Likert response scale. The PedsQL GI Symptoms Total Score (58-items) was calculated by reverse scoring and averaging responses from the ten GI-specific symptom scales (i.e., stomach pain and hurt, stomach discomfort when eating, food and drink limits, nausea and vomiting, gas and bloating, constipation, blood in poop, diarrhea, worry about going poop, worry about stomach aches). This yielded a measure of the impact of IBS-related symptoms on overall health and well-being across the last month, with lower scores reflecting greater impairments in quality of life. In addition, impairments in physical functioning and limitations due to symptoms were assessed using the FDI, a 15-item measure that asks participants’ to rate the level of difficulty associated with performing routine daily activities on a 5-point Likert scale ranging from 0 (“no trouble”) to 4 (“impossible”), with higher scores reflecting greater functional disability. The presence of mood disorders, including depression and anxiety was evaluated using the RCADS. The RCADS is a 47-item, self-report questionnaire with subscales that include separation anxiety disorder, social phobia, generalized anxiety disorder, panic disorder, obsessive-compulsive disorder, and major depressive disorder. It also yields a Total Anxiety Score (sum of the 5 anxiety subscales). Items are rated on a 4-point Likert-scale from 0 (“never”) to 3 (“always”). Lastly, the PCS-C is a 13-item questionnaire used to examine how catastrophic thinking is related to the experience of pain in children. The pain catastrophizing construct is comprised of three dimensions: rumination, magnification, and helplessness. Patients are asked to rate how strongly the thought or feeling prevails when experiencing pain. Items are rated from 0 (“not at all”) to 4 (“all the time”).

In addition, clinical outcome measures including disease duration (duration of IBS symptoms in yrs.) and pain intensity (over the past week) in relation to IBS symptoms were evaluated by a gastroenterologist at the time of the clinical visit, with the latter assessed verbally using a 0 to 10 numerical rating scale (NRS; 0 = no pain and 10 = worst pain imaginable).

### MRI Data Acquisition and Image Preprocessing

Imaging was performed with a Siemens 3 Tesla Trio Tim MRI scanner equipped with a 32-channel head coil. A high-resolution, T1-weighted magnetization-prepared rapid gradient-echo sequence was acquired for each participant [slices = 176, field of view = 220 x 220, echo time = 1.74, repetition time = 2520, flip angle = 7°, resolution = 1 x 1 mm, slice thickness = 1 mm, no gap]. The anatomical scan was followed by a functional T2-weighted echo-planar imaging scan during ‘resting-state’ wherein participant was instructed to relax with eyes open (slices = 34, 300 volumes, field of view = 224 x 224 mm, echo time = 30 ms, repetition time = 2010 ms, flip angle = 90°, resolution = 3.5 x 3.5, slice thickness = 5 mm). Diffusion tensor imaging and pseudo-continuous arterial spin labeling scans were also acquired following the resting-state functional MRI (fMRI) scan (data to be presented in a separate report). Total scan time was approximately 50 min.

The preprocessing pipeline for our cortical thickness analysis was performed using FreeSurfer (version 5.3.0) (http://surfer.nmr.mgh.harvard.edu), a semi-automated toolbox for reconstruction and visualization of the cortical surface [29–31]. The pipeline consisted of affine registration of the T1-weighted volume to Talairach space, skull stripping, white matter (WM) segmentation and tessellation of the gray/white matter boundary. At each step, visual inspection and manual correction of topological errors were performed. Following reconstruction of the cortical surface, brains were inflated, averaged across participants to produce a study-specific brain, and then smoothed using a 10 mm full-width at half maximum Gaussian kernel. Smoothing was performed on the surface tessellation using an iterative nearest-neighbor procedure, avoiding averaging of data across sulci or outside gray matter (GM). Each hemisphere was then parcellated into 34 distinct regions using the Desikan-Killany atlas [32]. A direct measure of cortical thickness was calculated using the shortest distance (mm) between the pial surface and gray-white matter boundary at each point or vertex of the cortical mantle, using spatial intensity gradients without limitation of individual voxel intensities, and therefore allowing for subvoxel resolution.

For the resting-state fMRI (rs-fMRI) data, all preprocessing was performed using Statistical Parametric Mapping version 8 (SPM8; Wellcome Institute of Cognitive Neurology, London) in Matlab (R2015a). Preprocessing steps included slice timing correction, motion correction, coregistration of the anatomical image to the mean functional image, segmentation of the anatomical image into GM, WM, and CSF, and normalization of anatomical and functional images to the standard Montreal Neurologic Institute (MNI) 152 brain template (voxel size = 2 mm^3^). Normalized images were then smoothed with an 8 mm isotropic full-width at half maximum Gaussian Kernel.

Single-subject first level resting-state functional connectivity (RS-FC) analysis was performed with the Functional Connectivity toolbox (CONN toolbox; version 15.g) in Matlab [33] (www.nitrc.org/projects/conn). Each subject’s warped anatomical and smoothed preprocessed functional images were specified along with regions of interest (ROIs) for GM, WM, CSF and seeds. At the first-level, covariate regressors were entered into the model including segmented WM and CSF, realignment parameters obtained during the motion correction preprocessing step in SPM8, and transient spikes identified in the fMRI time series representing motion outliers obtained from the Artifact Detection Tools software program (www.nitrc.org/projects/artifact_detect/). A denoising step was conducted using the principal components based ‘*aCompCor*’ method [34] which does not rely on global signal regression but instead identifies principal components from segmented WM and CSF [33, 35, 36]. Resting-state fMRI data were bandpass filtered (0.008 to 0.09 HZ) and signal associated with the six motion parameters, motion outliers, and those from WM and CSF seed regions were regressed from the fMRI time series.

### Statistical Analysis for Demographic, Psychometric and Clinical Measures

All statistical analyses for demographic, psychometric, and clinical measures were performed in SPSS version 21. Group comparisons using parametric or nonparametric tests were conducted as deemed necessary. To examine whether groups differed in age or eTIV, a series of one-way analysis of variance (ANOVAs) were performed. Additionally, a Chi-square (χ^2^) test of independence (Fisher’s Exact Test) was also conducted to examine whether groups differed proportionally for the gender variable. Differences in both age and gender were not expected since groups were matched on both variables. We also tested the extent to which psychometric and clinical measures were related using bivariate correlational analysis (Pearson’s r; 2-tailed) for the patient group.

### Surface-based Cortical Thickness Analysis

Cortical thickness analysis for each hemisphere was conducted using FreeSurfer’s Query, Design, Estimate, Contrast (QDEC) graphical interface (version 1.5). Group comparisons were performed using a general linear model (GLM; Design Matrix: Different Offset; Different Slope) with diagnosis specified as the fixed factor and age designated as a nuisance variable. Estimated total intracranial volume (eTIV) was not entered into the model as a nuisance variable since this metric has been shown to be both genetically and cellularly independent of cortical thickness unlike surface area which is known to depend on cortical volume [37–39]. Results for each analysis were overlaid onto the average pial and inflated surface maps using QDEC. An initial cluster forming significance threshold of *p* ≤ 0.005 with a cluster extent of 100 was used. Correction for multiple comparisons was performed using random-field-theory-based significant clusters at *p* < 0.05. Talairach coordinates for peak vertices yielding significant between-subject effects were converted to Montreal Neurological Institute (MNI) space using GingerALE (version 2.3.4) (www.brainmap.org/ale/). In addition, we also examined post-hoc, group differences in other surface-based metrics including surface area (pial) and cortical volume. For these analyses, eTIV was entered as a nuisance variable along with age since both variables are known to be potential confounders, especially during brain development. Results for surface area and cortical volume analyses were supplemental (S1 Table).

### Cortical Thickness, Psychometric and Clinical Variables

We performed a series of linear regression GLM analyses in QDEC (with age specified as a nuisance variable) to examine the relationship between cortical thickness and psychometric and clinical measures related to IBS-symptoms and clinical metrics, including abdominal pain severity (API), the impact of GI-specific symptoms on HRQOL (Total Symptoms scores from the PedsQL GI Symptoms Module), disease duration and pain intensity ratings, which were assessed at the time of the clinical visit. We also explored, post-hoc, the relationship between cortical thickness and functional disability (FDI), Total Anxiety (from the RCADS) and pain catastrophizing scores (PCS). Results for each analysis were overlaid onto the average pial and inflated surface using QDEC. An initial cluster forming significance threshold of *p* ≤ 0.005 and a cluster extent of 100 was used. Correction for multiple comparisons was performed using random-field-theory-based significant clusters at *p* < 0.05. Coordinates for peak vertices yielding significant between-subject effects were converted from Talairach to MNI space using GingerALE.

### Seed-based Resting-state Functional Connectivity Analysis

A series of seed-to-voxel whole brain analyses using 10 mm diameter spherical ROIs as seeds were performed in CONN toolbox. All seeds were generated in MarsBar (http://marsbar.sourforge.net) using peak voxel coordinates (i.e., MNI) for clusters demonstrating significant group differences in cortical thickness from the surface-based analysis. These seeds were all lateralized to the right hemisphere and included the dorsomedial prefrontal cortex (DMPFC: 19, 41, 43), the dorsolateral prefrontal cortex (DLPFC: 43, 27, 38), the posterior parietal cortex (PPC: 21, -57, 60) and the posterior cingulate cortex (PCC: 15, -29, 38). Two additional seeds (10 mm diameter spheres) were chosen based on significant negative correlations obtained between our cortical thickness analysis and measures of abdominal pain severity (API scores) and the impact of GI-specific symptoms on HRQOL (Total Symptom scores from the PedsQL GI Symptoms Module). These included seeds for the left anterior insula (aINS: -32, 21, -5) and the inferolateral cluster in the right primary somatosensory cortex (SI: 67, -10, 27).

At the second-level, a series of voxel-wise analyses were performed and functional connectivity maps obtained for each of the six seeds. An initial height threshold level of *p* < 0.005 was used. Correction for multiple comparisons was accomplished with 3dClustSim using Analysis of Functional NeuroImages (AFNI; http://afni.nimh.nih.gov/afni/) [40] software which calculated the minimum number of contiguous voxels required for a cluster to be deemed significant (2-sided threshold for t-tests) at an FDR of *p* < 0.05 (5000 iterations). The final functional connectivity maps were projected onto a study-specific brain generated by averaging all participants’ warped T1-weighted images using Ged Ridgway’s Masking toolbox in SPM8 (http://www0.cs.ucl.ac.uk/staff/g.ridgway/masking/) [41].

## Results

### Demographic and Sample Characteristics

Out of the 19 adolescent patients enrolled in the study, two were excluded from all statistical analyses; one patient was omitted due to being asymptomatic (no longer presenting with IBS symptoms) at the time of scanning, while another patient’s structural data was deemed unusable due to excessive motion artifacts. Thus, the final study sample consisted of 17 adolescents with IBS (Females = 13; mean age ± SD = 16.44 ± 1.73; range, 12.43–18.5) that were age and gender matched to 17 healthy cohorts (Females = 13; mean age ± SD = 16.29 ± 1.83; range, 11.73–20.3). The demographic distribution for ethnicity of the entire sample was 88.24% Caucasian (30/34), 8.82% Asian (3/34), and 2.94% African American (1/34) (IBS patients: Caucasian = 16/17; African American = 1/17; Asian = 0/17). One-way ANOVAs reveled no significant group differences between age [F (1,32) = 0.06, *p* = 0.8] or eTIV [F (1, 32) = 3.94, p = 0.06]. Furthermore, the Chi-square test showed no difference in the proportion of males to females between groups [χ^2^ (1) = 0, *p* = 1.0].

Data for individual patient characteristics such as age, gender, bowel habit subtype, disease duration, and severity measures (i.e., pain intensity) are shown in Table 1. Our patient sample was predominantly female and heterogeneous with regard to bowel habit subtype, with the majority reporting constipation predominant symptoms (IBS with constipation, IBS-C = 9, IBS with diarrhea, IBS-D = 5, mixed IBS, IBS-M = 2, unspecified IBS = 1).

**Table 1:** Individual Patient Characteristics.

Patient	Age (years)	Gender	Bowel Habit	Disease Duration (years)	Pain Intensity (NRS)
1	18.41	Female	IBS-C	2	10
2	16.43	Female	IBS-C	3	8
3	16.9	Female	IBS-D	1.5	7
4	13.28	Female	IBS-C	2	7
5	17.59	Male	IBS-D	5	7
6	17.07	Male	IBS-D	3.5	9
7	17.55	Female	IBS-C	1	7
8	16.46	Female	IBS-C	2	8
9	18.5	Female	IBS-U	2	8
10	15.86	Female	IBS-C	6	8
11	16.67	Female	IBS-M	5	8
12	12.43	Female	IBS-D	1.5	8
13	18.3	Female	IBS-C	6	10
14	16.87	Male	IBS-D	3	9
15	15.06	Female	IBS-M	1	9
16	14.65	Female	IBS-C	7	9
17	17.5	Male	IBS-C	10	8

Abbreviations: Numerical rating scale; IBS-C = constipation predominant IBS; IBS-D = diarrhea predominant; IBS-M = IBS mixed; IBS-U = IBS unspecified.

Descriptive statistics for psychometric and clinical variables for the entire patient sample are displayed in Table 2. Overall, patients had an average disease duration of 3.62 yrs. (SD ± 2.52; range, 1–10 yrs.) and reported their pain intensity associated with IBS symptoms in the moderate to severe range, with an average NRS of 8.24 (SD ± .97; range, 7–10 NRS).

**Table 2:** Descriptives for Psychometric and Clinical Measures in IBS Patients.

	IBS Patients		N
	Mean	SD	
**Disease Duration (yrs.)**	3.62	2.52	17
**Pain Intensity**	8.24	0.97	17
**Abdominal Pain Index**	2.70	0.74	15
**Functional Disability Inventory**	21.13	10.92	15
**PedsQL**	60.33	14.98	12
**PedsQL GI Module (Total Score)**	54.36	11.37	12
**PedsQL GI Module (Total Symptoms)**	48.67	12.89	12
Worry about poop	81.67	11.15	12
Worry about abdominal pain	52.08	33.78	12
**RCADS**			
Total Anxiety T Score	44.36	11.72	14
Total Anxiety Depression T score	47.07	12.38	14
Separation Anxiety T Score	49.64	12.16	14
General Anxiety T Score	39.43	6.61	14
Panic T Score	53.14	12.59	14
Social Phobia T Score	43.93	11.46	14
Obsessive-Compulsive T Score	42.29	11.32	14
Total Anxiety Depression Score	34.57	18.74	14
Total Depression Score	57.23	12.21	14
Total Anxiety Score	23.50	14.88	14
**PCS-C**	24.82	10.33	11
Rumination	10.73	3.32	11
Magnification	3.36	2.46	11
Helplessness	10.73	5.83	11

Abbreviations: IBS = irritable bowel syndrome; SD = standard deviation; N = Total number of subjects with questionnaire data; yrs. = years; PedsQL = Pediatric Quality of Life Inventory; PedsQL GI Module = Pediatric Quality of Life Inventory Gastrointestinal Symptoms Module; RCADS = Revised Children’s Anxiety and Depression Scale; PCS-C = Pain Catastrophizing Scale−Child version.

### Relationship between Psychometric and Clinical Measures

Summary of results for the bivariate correlational analysis are reported in the supplemental section (S2 Table). Briefly, in patients, functional disability (measured using the FDI) was positively correlated with Total Anxiety scores (r = .62, *p* = .02) from the RCADS. Conversely, the PedsQL was negatively correlated with FDI (r = -.73, *p* = .007) and Total Anxiety (r = -.79, *p* = .002) scores. The PedsQL was also negatively correlated with pain catastrophizing scores from the PCS (r = -.62, *p* = .04). These findings indicate that pediatric IBS patients reporting lower HRQOL, also reported greater functional disability associated with their symptoms, greater overall mood disturbances, and a greater propensity to catastrophize about their pain.

### Group Differences in Cortical Thickness

**Fig 1:**
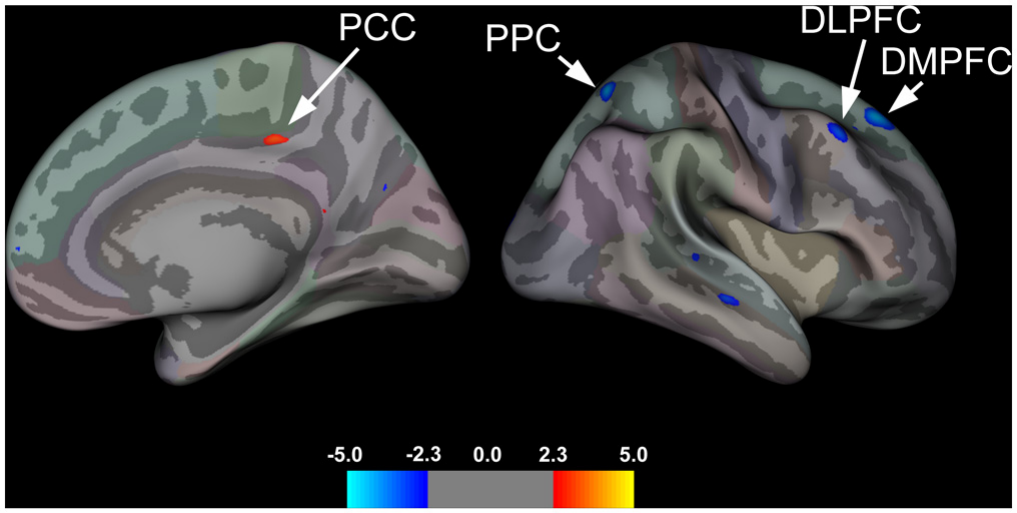
Group Effects for Cortical Thickness. Brain areas showing group differences in cortical thickness rendered onto the right hemisphere (medial and lateral view) of a semi-inflated averaged brain. Pediatric patients with IBS, compared to healthy cohorts, showed cortical thickening in the right posterior cingulate cortex (PCC) and cortical thinning in the right posterior parietal cortex (PPC), dorsomedial (DMPFC) and dorsolateral (DLPFC) prefrontal cortices.

A summary of group effects are displayed in Table 3 and illustrated in Fig 1. Relative to controls, pediatric patients with IBS showed significant cortical thickening in the right PCC, whereas cortical thinning in the right DMPFC, DLPFC, and PPC were found.

**Table 3:** Summary of Group Differences in Cortical Thickness.

Area	Side	NVtxs	Cluster Size (mm^2^)	Fvalue	MNI coordinates			Talairach coordinates		
					x	y	z	x	y	z
DMPFC	R	233	186.77	-4.1805	19.38	40.95	43.48	16.5	32.5	46.8
PPC	R	196	111.35	-4.1601	21.37	-57.28	60.44	17.9	-60.5	53.3
DLPFC	R	170	108.91	-3.3002	44.31	27.06	38.26	38.6	19.9	41.3
PCC	R	146	56.44	3.1398	15.18	-29.22	38.39	12.5	-32.4	36.0

Abbreviations: NVtxs = number of vertices; R = right; MNI = Montreal Neurological Institute; DMPFC = dorsomedial prefrontal cortex; PPC = posterior parietal cortex; DLPFC = dorsolateral prefrontal cortex; PCC = posterior cingulate cortex.

### Relationship Between Cortical Thickness and Abdominal Pain Severity

**Fig 2:**
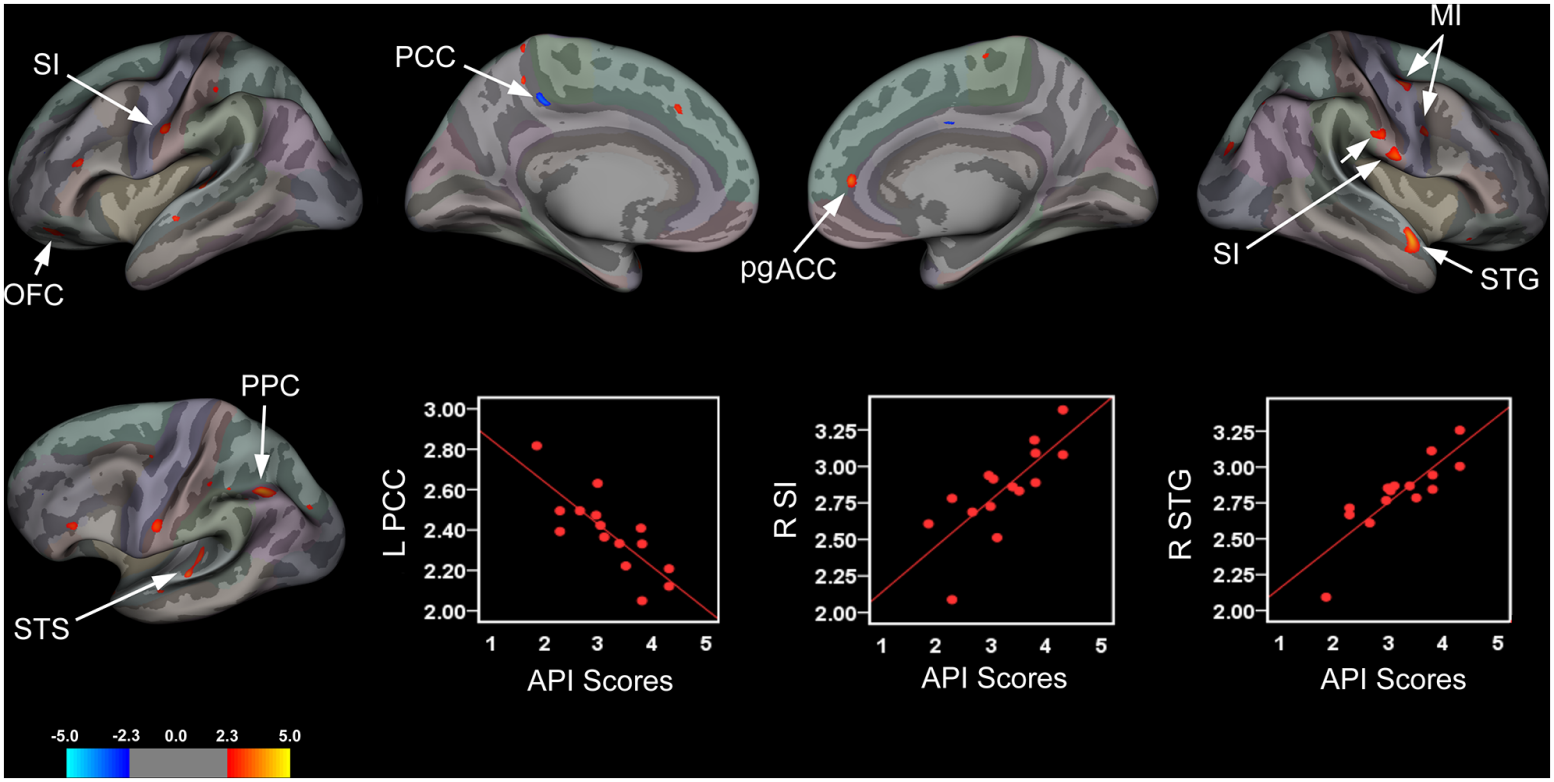
Brain Areas Showing Significant Associations between Cortical Thickness and Abdominal Pain Severity. Brain areas showing changes in cortical thickness that were associated with abdominal pain severity rendered onto an inflated averaged brain for the left and right hemispheres (lateral and medial views) with corresponding scatter plots for selected clusters. Patients with greater abdominal pain complaints exhibited significant cortical thinning in the left posterior cingulate cortex (PCC) and cortical thickening in the left orbitofrontal cortex (OFC), bank of the superior temporal sulcus (STS), and posterior parietal cortex (PPC). The latter two clusters are displayed on an inflated surface of the left hemisphere that was tilted approximately 15° to highlight areas exhibiting cortical thickening. In the right hemisphere, abdominal pain severity was associated with cortical thickening in the superior temporal gyrus (STG), primary motor cortex (MI), and perigenual anterior cingulate cortex (pgACC), although the latter cluster did not pass the defined cluster-extent threshold. Patients also showed cortical thickening in the bilateral primary somatosensory cortices (SI) corresponding to the intra-abdominal or ‘viscerotopic’ area, with two clusters in the right hemisphere; an the inferiorly- and a superiorly-placed cluster. Scatter plots depicting correlations between cortical thickness values (mm) for significant clusters in the left (L) PCC, right (R) inferior SI intra-abdominal area, and R STG with respective abdominal pain severity scores.

Surface-based analysis revealed significant associations between cortical thickness and abdominal pain severity as assessed by the API (Fig 2 and Table 4). In patients abdominal pain severity was correlated with cortical thinning in the left PCC and cortical thickening in the right DLPFC, superior temporal gyrus (STG), and two clusters in the right primary motor cortex (MI). In addition, greater severity of abdominal pain complaints was strongly correlated with cortical thickening in the left OFC, bank of the superior temporal sulcus (STS), PPC, and bilateral SI (Fig 2 and Table 4). With respect to the latter finding, more severe abdominal pain in patients was associated with cortical thickening localized to two distinct clusters located in the ‘intra-abdominal area’ of the right SI; an inferior and a superior cluster (Fig 2). Greater abdominal pain severity was also associated with cortical thickening in the right perigenual anterior cingulate cortex (pgACC; see Fig 2). Although this cluster passed the primary vertice-level threshold (*p* < .005), it did not survive the secondary cluster-extent threshold [F value = 3.5182, number of vertices = 63, cluster size = 35.83, MNI coordinates (x, y, z) = 16.02, 49.10, 60.88] and therefore will not be discussed further.

**Table 4:** Summary of Significant Clusters for Brain Areas Showing a Relationship between Cortical Thickness, Abdominal Pain Severity and Impact of GI-specific Symptoms on Health-Related Quality of Life.

		Side	NVtxs	ClusterSize (mm^2^)	F value	MNI coordinates		
						x	y	z
**Abdominal Pain Index**								
PPC (inferior parietal lobule)	L		347	173.72	3.9802	-36.74	-56.91	45.82
OFC	L		184	159.16	2.9112	-29.94	42.36	-19.03
STS	L		172	85.81	3.2437	-62.51	-26.22	5.8
STG	R		204	168.44	3.9534	53.11	-3.04	-23.57
SI	L		179	86.38	3.0979	-55.15	-8.95	27.05
SI (superior cluster)	R		205	65.13	3.3444	71.07	-7.47	10.74
SI (inferior cluster)	R		173	78.33	3.0020	66.64	-10.33	27.46
MI (superior cluster)	R		171	94.68	3.4159	45.89	-2.25	46.07
MI (inferior cluster)	R		100	52.47	3.2263	45.16	4.94	24.3
PCC	L		124	44.34	-3.3364	-14.6	-35.34	41.07
DLPFC	R		110	53.32	2.9164	29.53	1.15	43.21
**PedsQL GI Module (Total Symptoms)**								
STS	L		174	90.81	-3.2230	-53.11	-35.56	4.1
aINS	L		142	54.98	-4.4277	-31.66	21.19	-5.47
Fusiform	L		111	85.11	-3.3793	-30.04	-73.7	-7.88
PCC	R		83	42.46	-5.8281	6.88	-24.96	32.73

Abbreviations: NVtxs = number of vertices; R = right; L = left; MNI = Montreal Neurological Institute; PPC = posterior parietal cortex; OFC = orbitofrontal cortex; STG = superior temporal gyrus; SI = primary somatosensory cortex; MI = primary motor cortex; PCC = posterior cingulate cortex; DLPFC = dorsolateral prefrontal cortex; STS = superior temporal sulcus; aINS = anterior insula.

### Relationship Between Cortical Thickness and Health-related Quality of Life

**Fig 3:**
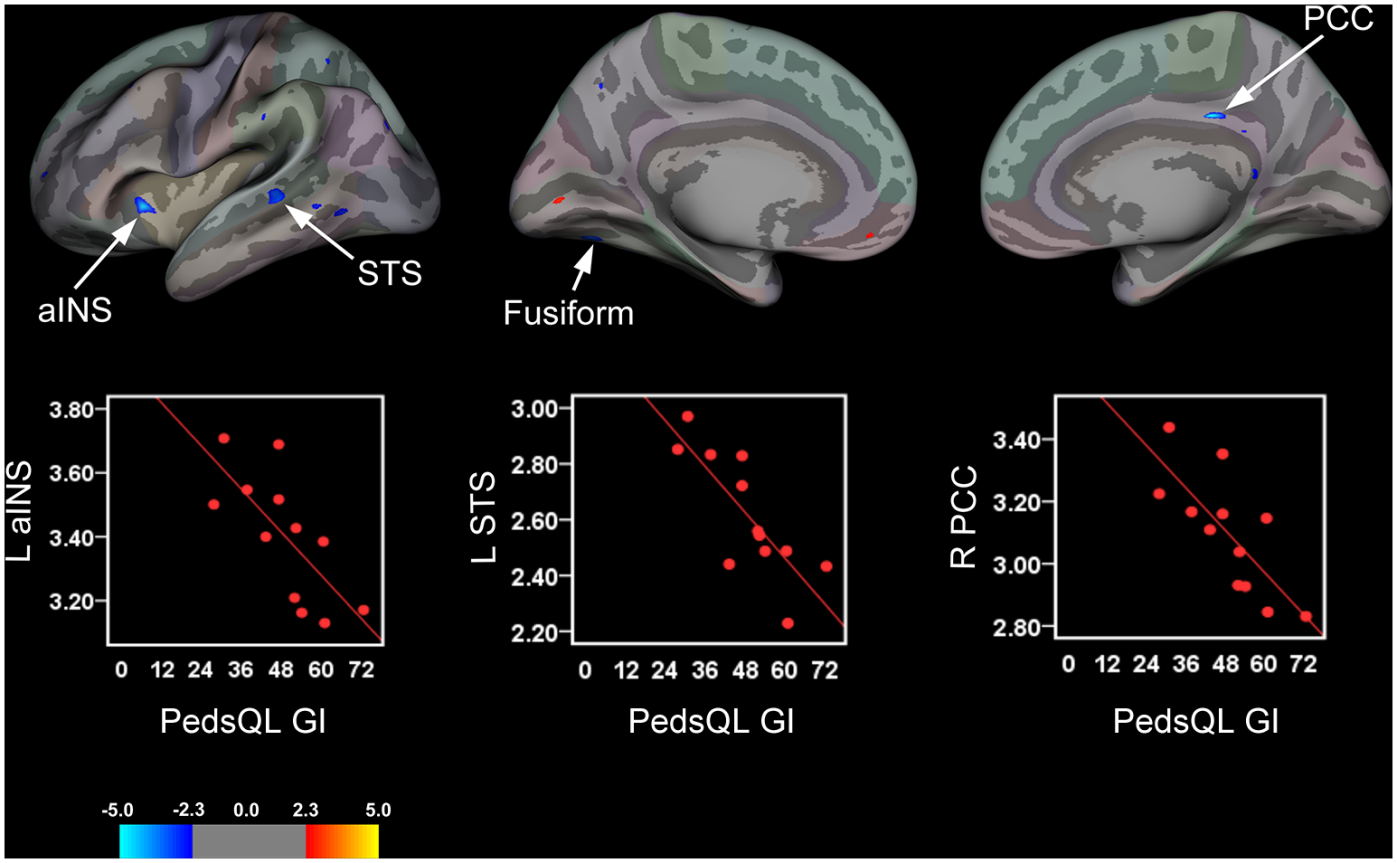
Brain Areas Showing Significant Associations between Cortical Thickness and Impact of GI-specific Symptoms on Health-related Quality of Life. Brain areas showing changes in cortical thickness that were associated with Total Symptoms scores from the PedsQL GI Symptoms Module rendered onto an inflated averaged study-specific brain for the left (lateral and medial views) and right hemisphere (medial view only) with corresponding scatter plots for selected significant clusters. For the PedsQL GI Symptoms Module (Total Symptom scores), greater reported HRQOL and lower GI-specific symptoms were associated with cortical thinning in the right (R) posterior cingulate cortex (PCC), left (L) anterior insula (aINS), L superior temporal sulcus (STS), and L fusiform gyrus.

Pediatric patients with greater HRQOL and reduced GI-specific symptoms (indexed by the PedsQL GI Total Symptom scores), exhibited significant cortical thinning in the right PCC and left anterior insula (aINS) (Fig 3 and Table 4). Negative associations were also observed between PedsQL GI Symptoms Module (Total Symptom scores) and cortical thickness in the left STS and fusiform gyrus

### Relationship Between Cortical Thickness and Clinical Measures

**Fig 4:**
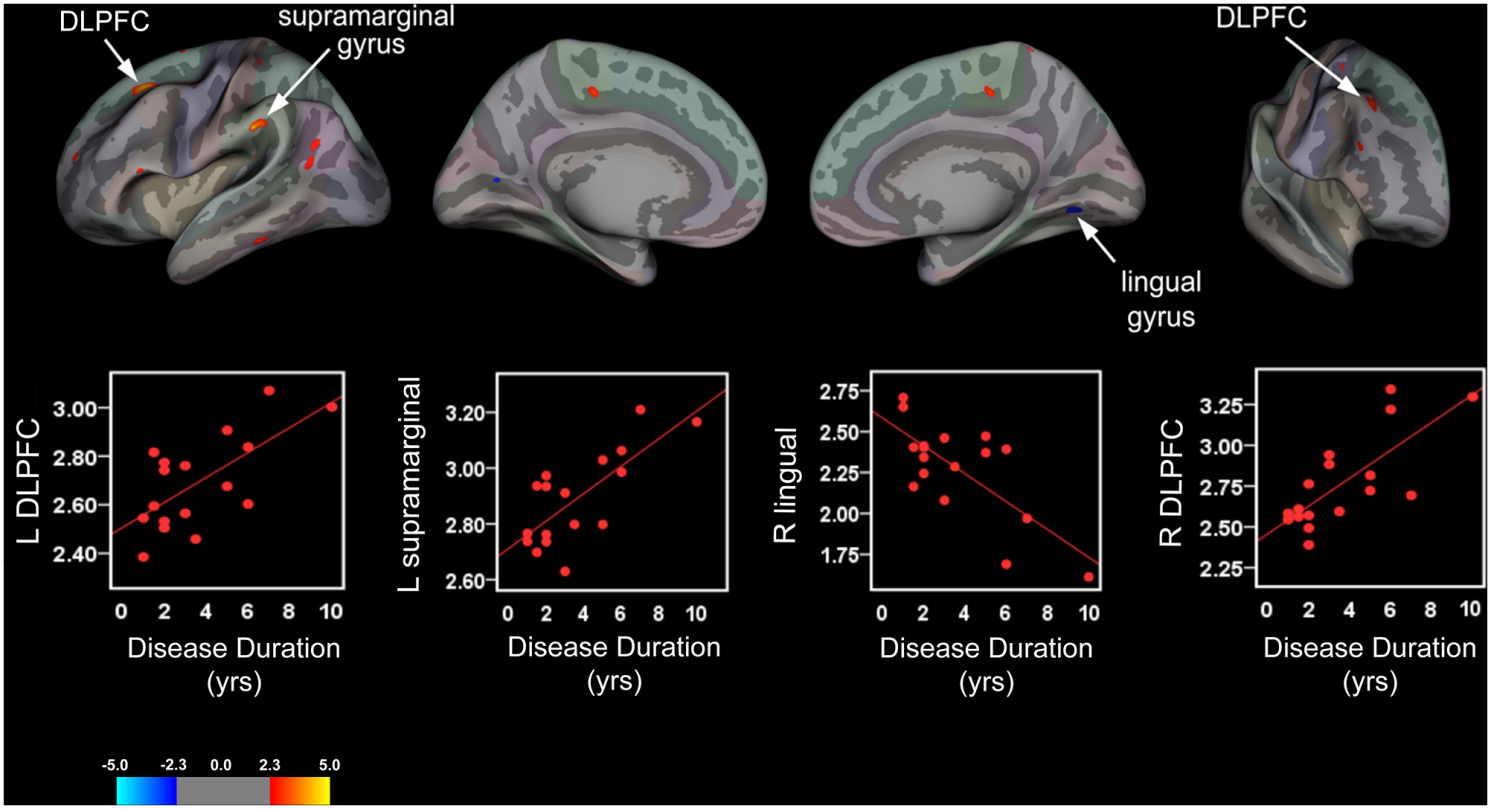
Cortical Thickness and Disease Duration in Pediatric IBS Patients. Brain areas showing a significant relationship between cortical thickness and disease duration rendered onto an inflated averaged study-specific brain for the left (L) and right (R) hemispheres (lateral and medial views) with corresponding scatter plots for selected clusters. Top panel: Longer disease durations (yrs) were correlated with cortical thickening (warm colors) in the L and R DLPFC, and L supramarginal gyrus. In contrast, cortical thinning (cool colors) in the R lingual gyrus was correlated with disease durations in IBS patients. Bottom panel: Scatter plots illustrating positive and negative correlations between cortical thickness values (mm) for significant clusters and duration of IBS symptoms.

**Fig 5:**
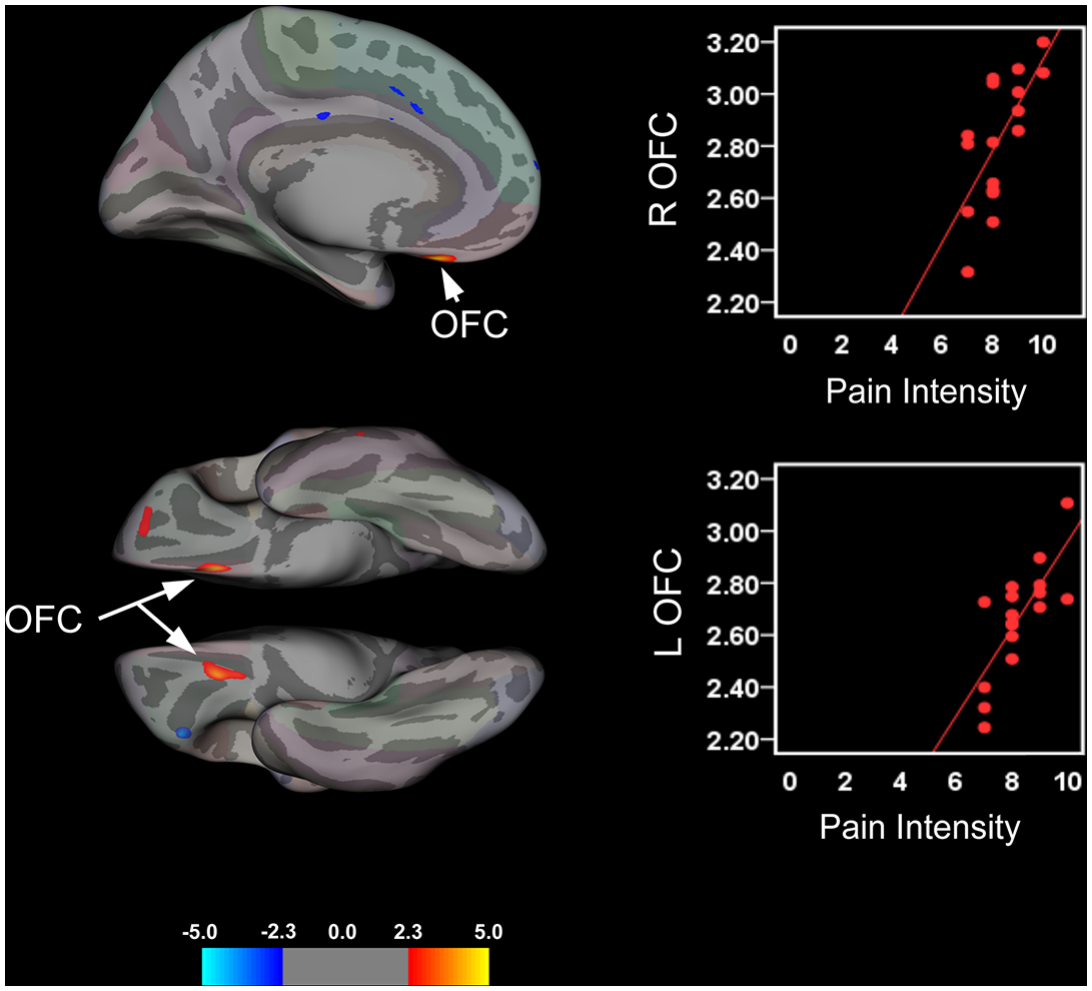
Cortical Thickness and Pain Intensity in Pediatric IBS Patients. Brain areas showing changes in cortical thickness that were associated with retrospective pain intensity ratings (over past week) rendered onto an inflated averaged study-specific brain with corresponding scatter plots for selected clusters. In patients, greater pain intensity ratings were correlated with cortical thickening in the bilateral orbitofrontal cortices (OFC). Top panel shows the medial aspect of the right hemisphere with a cluster in the OFC along with the corresponding scatter plot. Bottom panel shows the ventral aspect of the OFC for both hemispheres with scatter plot showing the relationship between cortical thickness and pain intensity ratings for the OFC cluster in the left hemisphere.

Results from the cortical thickness linear regression analysis with clinical variables are presented in Table 5 and illustrated in Figs 4 and 5, respectively. Analyses revealed significant positive correlations between disease duration and cortical thickness in bilateral DLPFC and left supramarginal gyri (Fig 4 and Table 5). A negative correlation between disease duration and cortical thickness in the right lingual gyrus was also found. Additionally, patients reporting higher levels of pain intensity associated with their IBS symptoms showed significant cortical thickening in bilateral OFC (Fig 5 and Table 5). Adolescent patients’ reporting greater pain intensity also exhibited cortical thinning in the left parsorbitalis and cortical thickening in the right MI.

**Table 5:** Summary of Significant Clusters for Brain Areas Showing a Relationship between Cortical Thickness, Disease Duration and Pain Intensity.

	Side	NVtxs	ClusterSize (mm^2^)	F value	MNI coordinates		
					x	y	z
**Disease Duration**							
Supramarginal gyrus	L	185	90.03	4.4821	-63.36	-32.31	31.4
	R	120	52.20	3.1117	57.56	-18	17.06
DLPFC	L	181	128.99	4.0740	-20.32	25.24	45.26
	R	116	77.36	3.0727	34.07	28.97	40.82
Lingual gyrus	R	110	110.07	-2.7540	18.94	-69.95	-4.82
**Pain Intensity**							
OFC	L	267	112.35	3.8616	18.17	21.53	-29.77
	R	194	98.54	4.6369	-6.06	23.94	-31.39
Parsorbitalis	L	110	92.47	-3.9611	46.85	36.44	-23.45
MI	R	195	79.54	2.9343	-46.42	-6.73	26.23

Abbreviations: NVtxs = number of vertices; R = right; L = left; MNI = Montreal Neurological Institute; DLPFC = dorsolateral prefrontal cortex; OFC = orbitofrontal cortex; MI = primary motor cortex.

### Relationship between Cortical Thickness, Functional Disability, Total Anxiety and Pain Catastrophizing

Our exploratory analysis revealed that patients’ functional disability scores as assessed by the FDI were positively correlated with cortical thickness in the right PCC and intraoral/face area of SI and the left lingual and supramarginal gyri. Conversely, decreased cortical thickness in left MI was associated with greater reported functional disability. For Total Anxiety scores, greater reported anxiety was associated with cortical thickening in the left PPC and supramarginal gyrus as well as in the right inferior temporal and fusiform gyri. We also observed a strong negative correlation between cortical thickness in the right DLPFC and pain catastrophizing. Due to the exploratory nature of these analyses and the lack of *a priori* hypotheses, we limit the presentation of these findings to the results section; however, supplementary information including a table (S3 Table) and figure (S1 Fig) are made available for the interested reader.

### Group Differences in Resting-state Functional Connectivity

**Fig 6:**
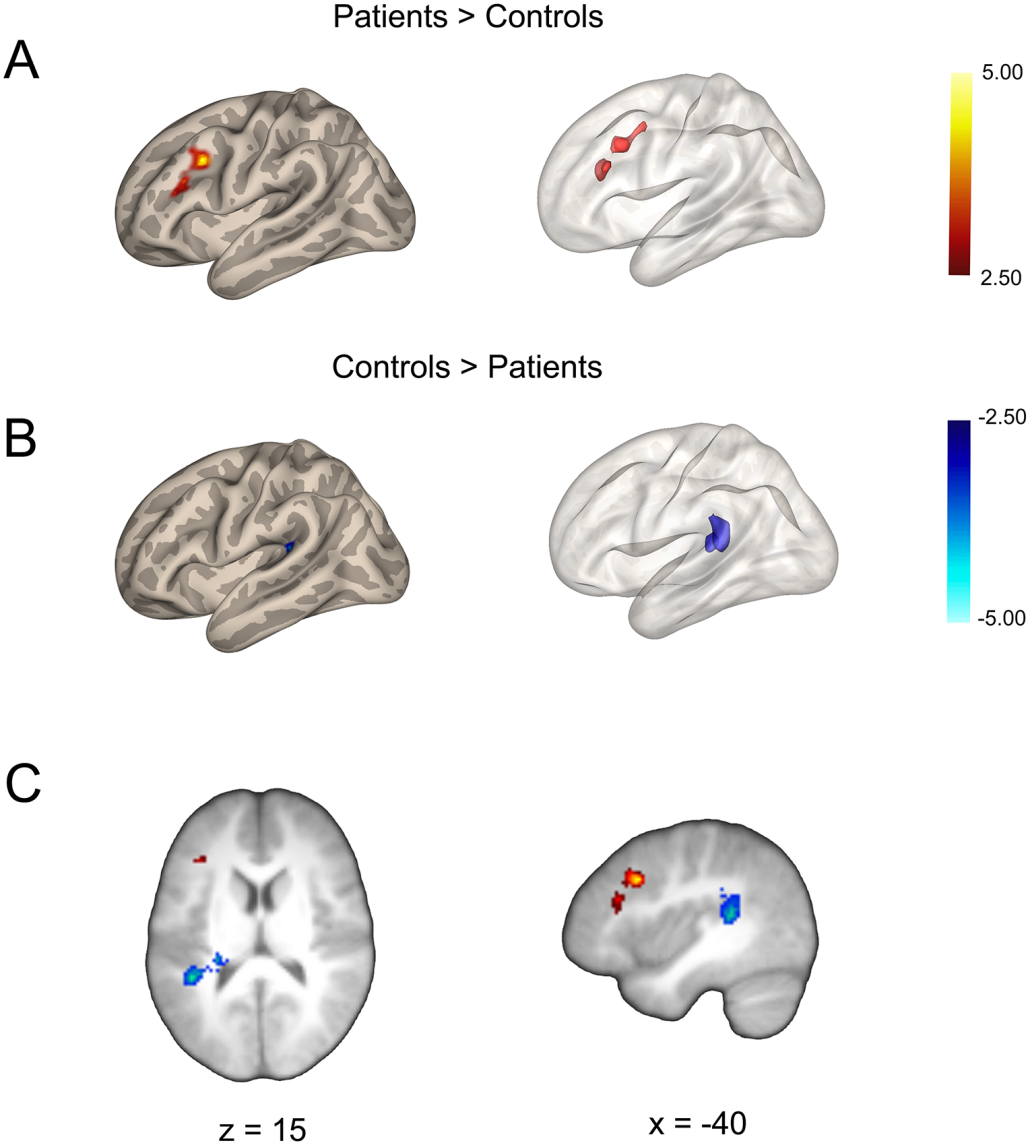
Group Effects for the Seed-based Functional Connectivity Analysis for the Posterior Cingulate Cortex. Seed-to-voxel whole-brain resting-state functional connectivity (RS-FC) results in pediatric IBS patients versus healthy controls for the left posterior cingulate cortex (PCC) seed rendered onto a semi-inflated brain showing cluster-corrected voxels (left panel) and the same cluster displayed in volume space rendered onto a semi-inflated glass brain (right panel) for the (A) dorsolateral prefrontal cortex (DLPFC) and the (B) parietal operculum. Statistical T maps showing results for RS-FC analysis for the PCC seed rendered onto the averaged study-specific brain (C). Hot voxels represent areas showing increased functional connectivity in patients relative to controls (Patients > Controls) for the PCC-DLPFC, whereas cool voxels represent decreased functional connectivity in patients versus controls for the PCC-Parietal Operculum (Controls > Patients).

We examined whether cortical areas showing structural brain changes in adolescents with IBS also showed functional impairments in intrinsic network connectivity. Results from our seed-to-voxel whole brain analysis are displayed in Table 6. No suprathreshold clusters were found for the DMPFC or PPC seeds for either contrast (i.e., patient > controls; controls > patient). For the right DLPFC seed, RS-FC analysis revealed increased connectivity in patients for a cluster with a peak voxel in right medial occipital lobe corresponding to the cuneus, and extending medially into the left hemisphere, whereas controls showed anti-correlated RS-FC patterning. Relative to healthy controls, pediatric patients with IBS showed greater connectivity between the right PCC seed and left DLPFC (Fig 6). Inspection of the normalized z-score connectivity values (Fisher transformed) revealed that the aforementioned result was due to an attenuation in anti-correlated RS-FC between the PCC-DLPFC in patients relative to controls, with controls showing increased anti-correlated RS-FC patterning compared to patients. In contrast, controls compared to patients showed greater connectivity or correlated patterning between the right PCC seed and the left parietal operculum (Fig 6). For the DLPFC seed, patients showed increased functional connectivity or correlated RS-FC with the right cuneus, whereas controls showed anti-correlated patterning. For L aINS seed, patients showed increased RS-FC compared to controls, for two large clusters in the occipital region with peak voxels in the right hemisphere. The largest cluster had a peak voxel placed medially, corresponding to the cuneus and which extended laterally into the right hemisphere and medially into the left hemisphere. The second smaller cluster also had a peak voxel in right lateral occipital cortex that was restricted to the right hemisphere. Interestingly, with respect to the aINS RS-FC, controls showed greater anti-correlations in both instances. Lastly, for the right SI seed that corresponded to the inferior portion of the intra-abdominal area, patients showed significant increases in correlated RS-FC compared to controls, whereas controls showed greater anti-correlations for a large cluster in the left cerebellum.

**Table 6:** Summary of Group Effects for Seed-based Functional Connectivity Analysis.

*Seed*	*Region*	*Side*	*Ke*	*Z value*	*T value*	*p*	*MNI coordinates*		
							x	y	z
**PAT > CTL**									
R DMPFC	No suprathreshold clusters								
R DLPFC	Cuneus	R	494	3.58	4.01	< 0.001	2	-94	6
R PPC	No suprathreshold clusters								
R PCC	DLPFC	L	330	4.01	4.62	0.002	-38	16	34
L aINS	Cuneus/Lateral Occipital Cortex	R	2109	3.95	4.53	< 0.001	10	-102	18
	Lateral Occipital Cortex	R	1218	3.42	3.79	< 0.001	28	-96	4
R SI^a^	Cerebellum	L	693	4.16	4.84	< 0.001	-2	-54	-32
**CTL > PAT**									
R DMPFC	No suprathreshold clusters								
R DLPFC	No suprathreshold clusters								
R PPC	No suprathreshold clusters								
R PCC	Parietal Operculum	L	338	4.73	5.74	0.002	-24	-32	10
L aINS	No suprathreshold clusters								
R SI^a^	No suprathreshold clusters								

Abbreviations: PAT = patients; CTL = controls; R = right; L = left; DMPFC = dorsomedial prefrontal cortex; DLPFC = dorsolateral prefrontal cortex; PPC = posterior parietal cortex; PCC = posterior cingulate cortex; aINS = anterior insula;

^a^SI = primary somatosensory cortex (intra-abdominal area), inferior cluster;

MNI = Montreal Neurological Institute.

## Discussion

While a number of neuroimaging studies exist in the extant adult IBS literature [15, 42], we are aware of only one report on functional imaging brain changes in a pediatric population and that is in response to liminal and subliminal rectal distention [43]. In adults with IBS, numerous structural and functional brain changes have been reported [15], including alterations in cortical thickness and gray matter volume [44–47], white matter integrity [48], and disturbances in intrinsic network connectivity [49, 50]. In contrast, little is known about the disease-related structural and functional brain changes that occur in the pediatric brain. Here, we report for the first time concomitant alterations in brain morphology and RS-FC patterns in adolescents who met Rome III criteria for IBS. The main findings observed were: (1) IBS patients, compared to a healthy cohort, showed significant cortical thickening in the right dorsal PCC, and cortical thinning in the right DMPFC, DLPFC and PPC; (2) In patients, structural alterations, including cortical thickening in the bilateral ‘viscerotopic’ portion of SI, were related to greater abdominal pain severity scores, whereas cortical thinning in the left aINS was associated with greater HRQOL and a reduction in GI-specific symptoms; (3) Clinical characteristics including disease duration and pain intensity (related to IBS symptoms) were correlated with cortical thickening in the DLPFC and OFC, bilaterally; and (4) During resting-state, patients exhibited less anti-correlated RS-FC compared to controls between the right PCC seed and the left DLPFC, indicating aberrant connectivity patterns in key nodes of the default mode and cognitive control networks. Together, these findings indicate extensive structural changes in the brains of pediatric IBS patients compared to healthy controls that encompassed areas important for higher-order cognitive functioning, as well as pain processing, interoceptive awareness and integration of viscerosensory information. A subset of these structural brain changes were correlated with disease severity measures and disrupted intrinsic network connectivity patterns. Understanding the structural and functional brain alterations that occur at earlier stages of this syndrome may provide novel insights into the evolution of IBS and possibly lay the groundwork for the development of future biomarkers that will enhance our ability to monitor brain changes following therapeutic interventions and that may precede the clinical/subjective benefits.

### Structural Brain Changes in Pediatric IBS

Our surface-based analysis revealed significant differences in cortical thickness in adolescents with IBS compared to healthy cohorts. Notably, patients showed a thickening in the right dorsal aspect of the PCC (dPCC), buried deep within the ventral bank of the posterior cingulate sulcus. The PCC is putatively involved in a number of processes [51]: (1) it is regarded as a core node of the default mode network (DMN) [52]; (2) it has been implicated in internally directed cognition [53] [54, 55]; (3) conscious awareness [56, 57]; (4) working memory [58]; (5) visuospatial orientation of the body in space or topokinesis [59], and (6) attention [60]. Moreover, there is a link between chronic pain conditions and abnormal task-dependent and intrinsic dPCC functioning. In visceral pain syndromes such as IBS increased activation in the dPCC to painful stimuli [61, 62], increased DMN connectivity with pain-related regions after lidocaine treatment [63] and diminished PCC functional connectivity during resting-state [64] have been found. The aforementioned functional changes in the PCC are in accord with our results demonstrating cortical thickening in this region in pediatric IBS patients. Although the causal factors underlying these disease-related structural changes in the dPCC cannot be determined here, they are unlikely due to simple age- or gender-related effects since IBS and healthy controls groups were matched on both variables. Further, age was controlled for in our analyses (specified as a nuisance variable). More parsimonious is an explanation that is viewed in context of the disease state. The cortical thickening of the dPCC in pediatric IBS patients observed in the present study may arise from a myriad of events that transpire as a result of IBS-related symptoms and progression of the disease over time. Alternatively, cortical thickness changes in the dPCC could result from different cortical developmental trajectories in patients relative to controls, wherein existing brain vulnerabilities or preconditions predispose certain individuals toward the development of aberrant structural brain changes during childhood and/or adolescence which then evolve and ultimately lead to the chronic disease state. Possible mechanisms for these neuroanatomical changes include increased dendritic aborization, spine density, synaptogenesis, unmasking of silent synapses, and/or use-dependent cortical restructuring (long-term potentiation; LTP) resulting from central sensitization of pathways conveying visceral nociceptive afferent signals from the gut to the brain.

In addition to increased cortical thickness in the dPCC, significant cortical thinning was also observed in pediatric IBS patients, relative to controls, in fronto-parietal areas such as the DMPFC, DLPFC, and PPC. The latter two areas comprise nodes of the cognitive control network (CCN) [65] and have been implicated in the integration of executive functions [66] such as attention [67], visuospatial working memory [68], decision-making, and cognitive inhibitory control processes [69], including descending pain modulation [70]. The DMPFC is also involved in mediating higher-order executive functions, but more in relation to social cognition, self-monitoring, affect regulation, and retrieval of autobiographical memories with social-emotional components. This area is also considered a key node of the DMN. All three of these areas share anatomical connections with the dPCC [54] [69, 71, 72]. Neuroimaging studies conducted in adults with IBS have shown disruptions in some of these same areas during both resting-state [64] and cognitively-demanding tasks [73], and in response to cued or uncued expectation of noxious stimulation to the abdomen [74]. In a previous study [75] we showed greater activation in the DMPFC in adult IBS patients compared to controls during an executive task used to assess global attentional network functioning. It is interesting to note that greater DMPFC activity was positively associated with GI symptom severity as well as pain catastrophizing and fear of uncertainty in these patients. The current findings demonstrating cortical thinning in the DMPFC in pediatric IBS patients may reflect the development of altered cognitive control functioning, and may over time, manifest as disruptions in corticolimbic inhibitory processes involved in attention or perhaps even lead to impairments in the ability to effectively regulate negative emotions and cognitions related to visceral sensations and symptoms (GI symptom specific anxiety).

In a study conducted by Seminowicz et al. (2010) gray matter volume (GMV) decreases were also observed in the PPC and rostral areas of the DLPFC (BA 46) in adult IBS patients compared to controls, whereas increases in the caudal portion of the DLPFC (BA 9) were found [47]. Thus, our cortical thickness changes in the DLPFC and PPC in pediatric IBS patients replicate, in part, their VBM findings. Numerous studies utilizing morphometric approaches have shown diminution of cortical thickness or GMV in prefrontal and posterior parietal areas such as the DLPFC and PPC in other chronic pain conditions including chronic low back pain [76–78], hip osteoarthritis [79] and migraine [80]. Furthermore, reversal in GMV reductions in the DLPFC following intervention with cognitive behavioral therapy [81] or hip replacement surgery [79] have also been reported and demonstrate the malleability of these morphological changes. In sum, the altered structural brain changes in fronto-parietal areas observed in the present study may reflect impairments in cognitive control functions previously reported in IBS patients [73, 82–86], and that may be evident early in evolution of this syndrome and possibly amenable to treatment.

### Relationship between cortical thickness, abdominal pain, and GI-specific symptoms

Another important finding to emerge from this study was that pediatric IBS patients reporting greater abdominal pain severity, assessed using the API, showed significant bilateral cortical thickening in the inferolateral areas of SI, corresponding to the intra-abdominal or ‘viscerotopic’ homunculus and extending into the intraoral area. Histochemical tract-tracing and extracellular recording studies performed in monkeys and cats have shown that the intra-abdominal area of SI receives viscero-viscero and viscero-somatic inputs from third-order neurons arising predominantly via the spinothalamic tract, however, dorsal-column medial lemniscal [87, 88] involvement has also been identified. Furthermore, human studies utilizing neuroimaging and evoked potential techniques have shown activation of the intra-abdominal area following stimulation to the upper and lower GI tracts [89–96]. Our findings of cortical thickening in the viscerotopic portion of SI in adolescent patients with IBS reporting greater abdominal pain are in accord with these results and are also consistent, in part, with a recent report by Irimia and colleagues [48] demonstrating greater mean fractional anisotropy (FA) in white matter connections to this area using diffusion tensor imaging in adult IBS patients. It should be noted that similar somatotopic findings have been reported for headache (SI; first division of the trigeminal representation of the face) suggesting a common underlying nociceptive drive in these conditions [97]. However, despite the fact that the locations for reported differences in WM connectivity as measured by mean FA overlap remarkably with clusters displaying increased cortical thickness reported here, Irmia et al. (2015) found no evidence of group differences in cortical thickness nor did they find any relationship between mean FA and a measure of usual IBS severity (assessed using the Bowel Symptom Questionnaire). This difference in findings may be due to the population studied since we investigated cortical thickness in pediatric patients with IBS, whereas their study was performed in adults. Other methodological differences include the measures administered to assess symptom severity. Regardless of these differences, we are the first to report structural brain changes coincident with a behavioral report of abdominal pain severity in pediatric IBS patients. These findings may shed new light on the cortical reorganization that accompanies behavioral changes specific to this syndrome. Other areas that showed positive correlations between abdominal pain severity and cortical thickness included bilateral MI, PPC, and superior temporal gyri/sulci, while a decrease in cortical thickness was identified in the dPCC. Although interpreting these findings with regard to the underlying etiology is complicated by the fact that causal relationships cannot be inferred, possibilities include but are not limited to changes in dendritic morphology previously mentioned above, including the recruitment and subsequent unmasking of so-called ‘silent nociceptors’, a premise put forth by Cervero and Jänig [98], and reviewed by Derbyshire [99]. For example, these changes could be due to the repetitive barrage of nociceptive visceral input, perhaps as a result of peripheral factors such as inflammation or ischemic events over time, which in turn, lead to the central sensitization of second-order afferents and synaptic remodeling in thalamic pathways providing input (via third-order neurons) to the intra-abdominal area of SI. Future work is needed to determine whether these morphological changes are a consequence or a causal factor driving the emergence of IBS symptoms.

We also identified cortical thinning in the left aINS and right dPCC that was associated with greater HRQOL and a lessening of GI-specific symptoms in pediatric patients. These findings are intriguing given the role that the insula and PCC plays in pain processing and interoceptive awareness and in context of previous neuroimaging studies demonstrating abnormal functioning of these areas in patients with IBS and other chronic pain conditions [43, 46, 61, 62, 64, 100–106]. However, our aINS findings are somewhat at odds with previous morphometric studies including results reported by Davis and others demonstrating cortical thinning in this subregion in adult IBS patients [101, 107]. For example, we found no significant group differences for the aINS in our sample of adolescent patients relative to controls, although there was a small cluster in the left aINS that did not pass threshold correction (F = 2.45, Cluster size = 4.89 mm^2^; NVtxs = 12), but this was in the opposite direction. Furthermore, Davis et al. did not examine cortical thickness in relation to behavioral indices, making direct comparisons between studies untenable. Jiang et al. (2013) did examine disease severity and other psychometric measures in adult patients versus controls in relation to cortical thickness, however they report no significant correlations between cortical thickness in the aINS and their disease severity measures. Others have reported aINS thickening that was associated with longer disease durations [45] in adult IBS patients. Given that this is the first study to examine and identify structural alterations in a pediatric IBS population, and in relation to a measure of health-related well-being in context of GI-specific symptoms, it is not surprising that our results differed with respect to the adult IBS literature. A possible explanation for the disparate findings reported here and previous studies conducted with adult IBS patients may be simply attributable to age and therefore, differences in brain developmental trajectories. Other alternate explanations include but are not limited to differences in length (disease duration) and severity of the disease state (see next section). Clearly, more studies are needed in a larger sample of pediatric patients at discrete developmental stages in order to begin to delineate the morphometric brain changes related to brain development and those driven by the time course and severity of the disease.

Additionally, our finding of cortical thickness changes in the superior temporal sulcus (STS) (cortical thinning) in relation to improved HRQOL and a lessening of GI-specific symptoms is also of interest. While little is known about the role of this (non-hippocampal) region of the temporal lobe in relation to chronic pain, previous reports indicate that there may be a consistent contribution to pain processing. Alterations in functional responses to noxious heat [97] in an overlapping area of the temporal lobe have been reported in migraine, which shares some similarities to IBS (viz., central sensitization, visceral-type pain). This region is purportedly involved in sensory discriminative functions, including speech and biological motion perception [108–110]. In addition to sensory discriminative functions, the STS has also been reported to be involved in processing nonverbal social cues and emotional perception [111, 112]. Given that the pathophysiology of IBS has been linked to impaired quality of life, psychosocial functioning, and emotional processing deficits such as GI-specific anxiety, alexithymia and depression, these changes are of clinical importance [13, 113, 114]. While the functional significance of these results are not yet known, the fact that they were related to improved HRQOL indicate that these brain-behavioral changes may be useful as a metric for gauging the efficacy of therapeutic interventions in this clinical population.

### Relationship between cortical thickness and disease-related clinical variables

Disease duration was correlated with cortical thickening in the bilateral DLPFC, an area which has been implicated in higher-order functions including cognitive control processes such as decision making, attention and descending pain modulation. It is noteworthy that cortical thickening in the supramarginal gyrus and cortical thinning in the lingual gyrus were also significantly related to duration of IBS symptoms. We have previously reported increased functional changes in the supramarginal gyrus in response to noxious heat in migraine patients and suggested a putative role for this region in pain may relate to cognitive interpretive processes [115]. This region has also been shown to be activated by both painful and non-painful aversive stimuli [116]. Thus, it is possible that these disease-related changes in brain architecture reflect a disruption in network processes associated with preservation of self in relation to perceived or actual harmful stimuli, although the exact meaning of these results remain to be determined.

In contrast to previous findings reported by Blankstein et al. (2010) demonstrating cortical thinning in the aINS associated with longer disease durations in adult patients with IBS, we found no significant relationship between cortical thickness and disease duration for this brain region [46]. However, we did find a small cluster in the right aINS that passed initial cluster thresholding, but failed to pass the cluster extent threshold determined *a priori* using 3dClustSim (F = 2.41, Cluster size = 5.77 mm^2^; NVtxs = 14). Nevertheless, the discrepancy in findings between our study conducted in adolescents and previous studies conducted in adults may relate to a number of factors, including differences in age, severity of IBS symptoms, and duration of disease. Clearly, the pediatric brain is not mature and therefore highly mutable in comparison to the adult brain. Although age was specified as regressor of no interest in the present study and therefore controlled for in the analysis, these differences in findings could be due to the developing nature of the pediatric brain relative to the more mature, adult brain. Moreover, as one might expect our adolescent IBS patients had much shorter disease durations (1–10 yrs.) compared to adult IBS patients (2–20 yrs.) sampled in the study by Blankstein et al. (2010) and this difference may underlie the observed differences in aINS cortical thickness since longer disease durations may produce more profound structural and functional brain changes that are not readily observed in the early stages of this syndrome. The notion that early brain changes may produce secondary effects with time has not been evaluated in adult or pediatric patients (viz., early vs. late onset IBS in either group), but should be an area of future study.

We also report an association between pain intensity scores and structural changes in the OFC. Specifically, patients reporting greater pain intensity associated with their IBS symptoms also showed significant cortical thickening localized to the bilateral OFC. The OFC is highly interconnected to limbic structures including areas involved in autonomic and stress-related physiological and behavioral responses [117]. This region receives visceral input from sensory areas as well as provides output to hypothalamic and midbrain structures involved in regulation of homeostatic functions [118, 119]. In addition, the OFC is considered important for regulation of affect, decision making [120], reward expectation, descending pain modulation [121, 122], integration of visceral sensorimotor information [117, 123] and interoceptive awareness [106]. Our findings of bilateral OFC in pediatric IBS are consistent with previous neuroimaging studies demonstrating functional and structural brain alterations in this region in adult IBS patients. For example, Jarcho et al. (2008) showed symptom improvement with a 5-HT_3_ receptor antagonist that was predicted by attenuated activity in the bilateral OFC and left medial temporal gyrus in response to rectal distention prior to drug treatment [124]. Additionally, Piché et al. (2009) showed cortical thickening in the OFC predicted reductions in pain inhibition in both IBS patients and healthy controls [122]. Using VBM, Seminowicz et al. (2010) also reported increased gray matter density in the left OFC in adult IBS patients compared to controls, indicating that these changes may be common across age for the disease state.

### Functional Brain Changes in Pediatric IBS

Here we used resting-state fMRI and the regions noted above, that exhibited significant group differences in cortical thickness presumably due to underlying pathophysiology of IBS, as seeds in our RS-FC analysis (viz., PCC, DMPFC, DLPFC, and PPC). We also examined seeds in areas that showed structural changes that correlated with measures of abdominal pain severity and GI-specific symptoms in relation to HRQOL in the right intra-abdominal area of SI and the left anterior insula. In this way insights into how structural changes may be linked to reorganization of functional brain networks can be ascertained, although causal inferences still cannot be made.

Our seed-based analysis revealed altered RS-FC within key nodes of the DMN and CCN networks in adolescent patients with IBS. Specifically, we identified significant differences in RS-FC between the right PCC seed and the left DLPFC in patients relative to healthy cohorts of similar age. Inspection of the data revealed that this difference in RS-FC was not simply due to greater increases in RS-FC between the PCC-DLPFC in patients versus controls, but rather resulted from a diminution in anti-correlated connectivity patterns in the former group relative to the latter. Previous reports of alterations in canonical nodes of intrinsic connectivity networks such as the DMN have been described in clinical pain conditions [84, 125–128], including adults and adolescents with IBS during resting-state [64] and liminal rectal distension [43]. Structural and functional connections between the PCC and medial temporal and prefrontal (mPFC) and the inferior parietal cortices [129] have also been described using streamline and probabilistic tractography, and RS-FC methods [130]. Furthermore, the DLPFC has been implicated in chronic pain across numerous studies [70, 76, 81, 131, 132] and altered PCC-DLPFC functioning has been reported in fibromyalgia and migraine [84], suggesting that this area is involved across a spectrum of chronic pain conditions. The consequences of such changes in PCC-DLPFC connectivity could include alterations in attention [75, 133–137], arousal [54, 138] and cognitive control functions [139–141], a finding frequently reported for various chronic pain conditions. Although the exact functional significance of our RS-FC analysis are not known, the atypical connectivity patterns consisting of reduced PCC-DLPFC anti-correlations in patients compared to controls may reflect an attentional shift [54] of cognitive control areas toward viscerosensory and interoceptive processes related to chronic, ongoing abdominal pain and/or discomfort in these patients.

### Study Caveats and Limitations

Several limitations should be mentioned in context of the present study findings. First, our sample of adolescents with IBS diagnosis was heterogeneous with respect to bowel habit subtype, with the majority of patients classified as constipation predominant, therefore limiting the generalizability of our results. A second major limitation was that medications with psychotropic effects were not controlled for in the patient group and this could have negatively influenced our results. However, it should be noted that inclusion of patients on psychotropic medications was restricted to those patients on low to moderate, stable doses. Third, a lack of psychometric measures for the healthy control group limits our ability to determine whether depression or anxiety influenced our results since we had little to no data with regard to these metrics between groups and therefore could not control for these variables in our analyses. Lastly, our study design was cross-sectional and therefore prevents us from making causal inferences. Future studies utilizing a longitudinal design in pediatric IBS patients are needed in order to determine whether the observed alterations in brain architecture and intrinsic network functioning are a consequence of IBS symptoms over time, or represent an antecedent, premorbid brain state that renders the brain vulnerable to the development of gut-brain signaling abnormalities, which in turn may lead to IBS.

## Conclusions

In the present study, we evaluated concurrent structural and functional brain changes in adolescents with a diagnosis of IBS relative to a healthy cohort. We report both increases and decreases in cortical thickness in areas involved in cognitive inhibitory control, pain-related processes, viscerosensory motor and interoceptive functions, that were correlated with measures of abdominal pain severity, HRQOL in relation to GI-specific symptoms and disease severity measures such as duration of IBS symptoms and pain intensity. In addition, we also found atypical intrinsic RS-FC patterns between DMN and cognitive control network nodes, a finding which may reflect a shifting of attentional resources towards viscerosensory and interoceptive processes related to ongoing, abdominal pain. Whether these structural and functional brain changes are disease-driven or antecedent to the onset of childhood IBS remains to be determined. These brain changes (sensory, cognitive and emotional) may have long-term consequences on future behavior (e.g., anxiety, depression, psychosocial functioning, propensity to develop chronic pain, etc.), health and well-being, of individuals affected by IBS during their childhood years.

## Supporting Information

S1 Fig Pain catastrophizing was associated with cortical thinning in the right dorsolateral prefrontal cortex (DLPFC). (TIF)

S1 Table Group differences in surface area and cortical volume in pediatric patients with IBS versus healthy controls. Abbreviations: NVtxs = number of vertices; R = right; L = left; PPC = posterior Parietal Cortex; PCC = posterior cingulate cortex; SI = primary somatosensory cortex; MNI = Montreal Neurological Institute.
(DOCX)

S2 Table Summary of correlations between psychological measures and clinical disease characteristics for pediatric patients with IBS. Abbreviations: PedsQL = Pediatric Quality of Life Inventory; PedsQL GI Module = Pediatric Quality of Life Inventory Gastrointestinal Symptoms Module; API = Abdominal Pain Index; FDI = Functional Disability Inventory PCS-C = Pain Catastrophizing Scale−Child version.
(DOCX)

S3 Table Summary of significant clusters for brain areas showing a relationship between cortical thickness and functional disability, total anxiety, and pain catastrophizing. Abbreviations: NVtxs = number of vertices; R = right; L = left; MNI = Montreal Neurological Institute; FDI = Functional Disability Inventory; SI = primary somatosensory cortex; PCC = posterior cingulate cortex; PPC = posterior parietal cortex; PCS-C = Pain Catastrophizing Scale−Child version; DLPFC = dorsolateral prefrontal cortex.
(DOCX)
