# Supplementary figures and images for: Abdominal Pain, the Adolescent and Altered Brain Structure and Function
Source: PLoS One. 2016 May 31;11(5):e0156545. doi: 10.1371/journal.pone.0156545 (PMC4886967; doi:10.1371/journal.pone.0156545)

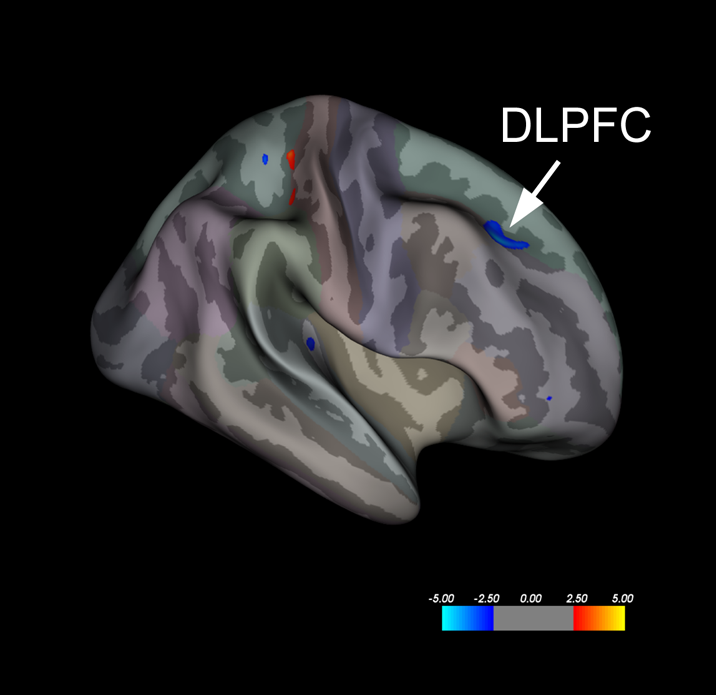

Supplement: S1 Fig — (TIF) [file pone.0156545.s001.tif]
