# Supplementary material for: Abdominal Pain, the Adolescent and Altered Brain Structure and Function
Source: PLoS One. 2016 May 31;11(5):e0156545. doi: 10.1371/journal.pone.0156545 (PMC4886967; doi:10.1371/journal.pone.0156545)
Supplement: S1 Table — Abbreviations: NVtxs = number of vertices; R = right; L = left; PPC = posterior Parietal Cortex; PCC = posterior cingulate cortex; SI = primary somatosensory cortex; MNI = Montreal Neurological Institute. (DOCX) [file pone.0156545.s002.docx]

|  | **Side** | **NVtxs** | **Cluster Size (mm^2^)** | **F value** | **MNI coordinates** | | |
| --- | --- | --- | --- | --- | --- | --- | --- |
|  |  |  |  |  | x | y | z |
| **Surface Area** |  |  |  |  |  |  |  |
| Middle temporal gyrus | R | 150 | 116.72 | -3.1301 | 57.03 | -23.25 | -17.26 |
| Cuneus | R | 158 | 128.41 | -4.1493 | 7.81 | -80.65 | 35.2 |
| Precuneus | R | 393 | 164.89 | -3.2292 | 25.52 | -59.9 | 12.67 |
| PPC | R | 681 | 508.39 | -3.5499 | 25.18 | -83.56 | 23.76 |
| Supramarginal gyrus | R | 107 | 45.23 | -3.0579 | 65.98 | -34.3 | 38.59 |
| Supramarginal gyrus | R | 172 | 75.23 | -2.7783 | 44.71 | -31.4 | 40.9 |
| Pericalcarine | R | 219 | 183.07 | -2.8982 | 16.34 | -80.68 | 9.06 |
| Lateral occipital gyrus | L | 263 | 189.98 | 5.0528 | -42.6 | -79.54 | -3.05 |
| Cingulate isthmus | L | 227 | 63.10 | -4.3155 | -8.04 | -46.65 | 10.48 |
| Parstriangularis | L | 167 | 130.71 | 2.9612 | -47.21 | 38.11 | -9.57 |
| SI | L | 129 | 55.66 | 2.8665 | -48.3 | -9.96 | 13.58 |
| **Cortical Volume** |  |  |  |  |  |  |  |
| Cuneus | R | 175 | 145.31 | -3.7959 | 7.58 | -80.1 | 34.14 |
| Precuneus | R | 246 | 110.14 | -3.3505 | 25.52 | -59.9 | 12.67 |
| PPC | R | 550 | 408.69 | -3.1946 | 13.11 | 44.04 | 43.61 |
| Middle temporal gyrus | R | 305 | 237.35 | -4.0673 | 60.68 | -20.24 | -18.52 |
| Supramarginal gyrus | R | 249 | 107.58 | -2.8345 | 45.69 | -31.13 | 41.53 |
| Lateral occipital gyrus | L | 233 | 167.84 | 6.6272 | -42.6 | -79.54 | -3.05 |
| Inferior parietal cortex | L | 257 | 118.61 | -3.2312 | -34.04 | -50.28 | 38.51 |
| Lingual gyrus | L | 188 | 109.68 | -2.7889 | -18.97 | -68.82 | 4.68 |
| Insula | L | 105 | 38.19 | 2.7063 | -28.02 | 22.49 | 0.28 |
