# Supplementary material for: Abdominal Pain, the Adolescent and Altered Brain Structure and Function
Source: PLoS One. 2016 May 31;11(5):e0156545. doi: 10.1371/journal.pone.0156545 (PMC4886967; doi:10.1371/journal.pone.0156545)
Supplement: S2 Table — Abbreviations: PedsQL = Pediatric Quality of Life Inventory; PedsQL GI Module = Pediatric Quality of Life Inventory Gastrointestinal Symptoms Module; API = Abdominal Pain Index; FDI = Functional Disability Inventory PCS-C = Pain Catastrophizing Scale−Child version. (DOCX) [file pone.0156545.s003.docx]

|  | **PedsQL** | **PedsQL GI Symptoms** | **API** | **FDI** | **Total Anxiety** | **PCS** | **DiseaseDuration** |
| --- | --- | --- | --- | --- | --- | --- | --- |
| **PedsQL GI** | .48 (.12) |  |  |  |  |  |  |
| **API** | -.45 (.14) | -.28 (.38) |  |  |  |  |  |
| **FDI** | **-.73 (.007)** | -.22 (.50) | .00 (.99) |  |  |  |  |
| **Total Anxiety** | **-.79 (.002)** | -.40 (.20) | .36 (.21) | **.62 (.02)** |  |  |  |
| **PCS** | **-.62 (.04)** | -.02 (.48) | .47 (.15) | .37 (.11) | **.56 (.07)** |  |  |
| **Disease Duration** | .30 (.34) | .025 (.94) | -.23 (.21) | .09 (.75) | .10 (.37) | -.59 (.06) |  |
| **Pain Intensity** | .17 (.52) | -.061 (.43) | -.06 (.85) | -.16 (.57) | -.04 (.57) | -.03 (.94) | .17 (.52) |
