# Supplementary material for: Abdominal Pain, the Adolescent and Altered Brain Structure and Function
Source: PLoS One. 2016 May 31;11(5):e0156545. doi: 10.1371/journal.pone.0156545 (PMC4886967; doi:10.1371/journal.pone.0156545)
Supplement: S3 Table — Abbreviations: NVtxs = number of vertices; R = right; L = left; MNI = Montreal Neurological Institute; FDI = Functional Disability Inventory; SI = primary somatosensory cortex; PCC = posterior cingulate cortex; PPC = posterior parietal cortex; PCS-C = Pain Catastrophizing Scale−Child version; DLPFC = dorsolateral prefrontal cortex. (DOCX) [file pone.0156545.s004.docx]

|  | **Side** | **NVtxs** | **ClusterSize (mm^2^)** | **F value** | **MNI coordinates** | | |
| --- | --- | --- | --- | --- | --- | --- | --- |
|  | | | | | x | y | z |
| **FDI** |  |  |  |  |  |  |  |
| SI | R | 233 | 115.49 | 3.2574 | 44.47 | -24.34 | 48.72 |
| PCC | R | 106 | 45.44 | 3.1671 | 8.22 | -30.62 | 43.24 |
| Lingual gyrus | L | 111 | 94.84 | 4.1155 | -21.18 | -66.35 | -6.62 |
| Supramarginal gyrus | L | 206 | 105.91 | 4.0092 | -51.01 | -48.98 | 38.44 |
| Precentral gyrus | L | 174 | 60.97 | -2.6818 | -4.68 | -23.8 | 75.62 |
| **Total Anxiety T Score** |  |  |  |  |  |  |  |
| Inferior temporal gyrus | R | 168 | 124.01 | 3.3424 | 47.56 | -13.27 | -37.36 |
| Fusiform | R | 113 | 74.36 | 3.1247 | 30.26 | -55.19 | -12.98 |
| PPC | L | 215 | 109.35 | 4.4598 | -27.37 | -41.53 | 72.04 |
| Supramarginal gyrus | L | 117 | 40.48 | 3.3775 | -45.16 | -26.4 | 36.22 |
| **PCS-C** |  |  |  |  |  |  |  |
| DLPFC | R | 271 | 188.74 | -4.1756 | 31.29 | 30.5 | 34.44 |
